# Influence of Omega-3 Fatty Acid-Rich Fish Oils on Hyperlipidemia: Effect of Eel, Sardine, Trout, and Cod Oils on Hyperlipidemic Mice

**DOI:** 10.1089/jmf.2020.0114

**Published:** 2021-07-19

**Authors:** Martha Kontostathi, Sofia Isou, Dimitrios Mostratos, Vassilios Vasdekis, Nikolaos Demertzis, Angeliki Kourounakis, Andreas Vitsos, Maria Kyriazi, Dimitrios Melissos, Charilaos Tsitouris, Evangelos Karalis, Lykourgos Klamarias, Fotini Dania, Georgios-Theodorou Papaioannou, Vassilios Roussis, Evangelos Polychronopoulos, Jane Anastassopoulou, Theophilos Theophanides, Michail-Christou Rallis, Homer S. Black

**Affiliations:** ^1^ Section of Pharmaceutical Technology, Department of Pharmacy, School of Health Sciences, National and Kapodistrian University of Athens, Athens, Greece.; ^2^ Department of Statistics, Athens University of Economics and Business, Athens, Greece.; ^3^ Section of Pharmaceutical Chemistry, Department of Pharmacy, School of Health Sciences, National and Kapodistrian University of Athens, Athens, Greece.; ^4^ Quality Assurance and Control Systems—QACS Lab, Athens, Greece.; ^5^ Klms Veterinary Clinic, Athens, Greece.; ^6^ Section of Pharmacognosy and Chemistry of Natural Products, Department of Pharmacy, School of Health Sciences, National and Kapodistrian University of Athens, Athens, Greece.; ^7^ Department of Nutrition and Dietetics, School of Health Science and Education, Harokopeion University, Athens, Greece.; ^8^ Radiation Chemistry and Biospectroscopy, Chemical Engineering School, National Technical University of Athens, Athens, Greece.; ^9^ Department of Dermatology, Baylor College of Medicine, Houston, Texas, USA.

**Keywords:** anti-hyperlipidemia effect, dyslipidemia, fish oils, high fat diet, omega-3 fatty acids

## Abstract

Dyslipidemia is one of the most important cardiovascular disease (CVD) risk factors. Polyunsaturated fatty acids (FAs), and especially omega-3 FAs, could significantly contribute to the management of dyslipidemia and the prevention of CVD. The anti-hyperlipidemic effect of selected fish oils (eel, sardine, trout, cod liver) was comparatively evaluated in a high fat diet (HFD)-fed mouse model. At the end of 30 days on the HFD, all animals were hyperlipidemic and were switched to a diet consisting of 90% standard rodent chow plus 10% of oil from eel, sardine, cod liver, or trout. At the end of 60 days on these diets, blood glucose, total blood cholesterol, triglycerides (TGs), and high density lipoprotein (HDL) were quantitated. All diets, except sardine and standard rodent chow, showed statistically significant decreases in blood glucose from day 30 to 90. Total blood cholesterol decreased in all diets except the HFD group, which was continued on this diet until the end of the study. Eel and cod liver oil diets showed significant decreases in TGs. All dietary groups showed a decrease in HDL, but only the trout and standard chow groups exhibited statistically significant decreases. The fish oils tested here for effects on hyperlipidemia vary in per cent of omega-3 FAs and omega-6/-3 FA ratios as determined by gas chromatography Overall, smoked eel was the best source of omega-3 FA, with a balance of omega-6 FA, that ameliorated HFD-induced mixed hyperlipidemia.

## Introduction

Cardiovascular disease (CVD) is one of the main causes of premature death worldwide.^1,2^ Dyslipidemia is one of the most important CVD risk factors,^3^ and it is characterized by the elevated concentration of circulating lipids.^2,4^ Its prominent role in the etiopathogenesis of atherosclerosis and other symptoms connected to CVD is well known.^5,6^ There are three distinct types of dyslipidemia: hypercholesterolemia (high cholesterol concentration), hypertriglyceridemia (high triglyceride [TG] concentration), and mixed hyperlipidemia (high cholesterol and TGs).^7^

Lifestyle modifications and pharmacotherapy are the prevailing approaches to manage dyslipidemia and, therefore, reduce CVD risk.^5,8^ Independent organizations have developed several guidelines for dyslipidemia management aiming at improving health care and reducing treatment costs.^5^ Nutritional factors play a key role in the prevention of CVD and of other metabolic events.^8^ The replacement of saturated by polyunsaturated fatty acids (PUFAs) in the diet reduces the CVD risk.^9^ PUFA can be divided into three different categories of fatty acids (FAs): omega-3 (*ω*-3), omega-6 (*ω*-6), and omega-9 (*ω*-9).^9^ The ratio *ω*-6/*ω*-3 seems to be a key factor in CVD prevention.^10^

Many epidemiological studies confirm the *ω*-3 FAs' positive impact on CVD, mainly due to their antithrombotic, anti-inflammatory, anti-arrhythmic, and TG reducing properties. The most common dietary *ω*-3 FAs are docosahexaenoic acid (DHA), eicosapentaenoic acid (EPA), and *α*-linolenic acid.^9^

Thanks to their protective effects, the *ω*-3 FA intake has increased worldwide.^4,11,12^ Numerous studies have shown that one out of five inhabitants of the Western world systematically consume supplements derived from fish oils, as they claim to provide anti-inflammatory effects against CVD and arthritis. Oils extracted from fishes, such as salmon, trout, sardine, herring, mackerel, and some mollusks, mainly contain *ω*-3 FA (EPA, DHA) whereas oils derived from fish liver show high confounding levels of vitamins A and D. Taking into consideration these benefits of selected fish oils, the National Institute for health and Care Excellence (NICE) in the United Kingdom encourages patients, especially those with a poor daily intake of *ω*-3 FA, to consume fish-oil supplements during the first 3 months of the heart attack recovery period.^11–13^ Indeed, in a recent population-based prospective cohort study involving almost a half million participants, habitual fish oil supplementation was found to be associated with a 13% lower risk of all-cause mortality, a 16% lower risk of CVD mortality, and a 7% lower risk of CVD events.^14^

The aim of this study was to evaluate and compare the anti-hyperlipidemic effects of selected fish oils (trout, sardine, eel, and cod liver) that are rich in *ω*-3 FA. The effects of fish oil on mouse serum lipids (cholesterol, TGs, high density lipoprotein [HDL], and glucose levels) were evaluated in a nutrition experiment on mice, after administration of a high fat diet (HFD) by which a hyperlipidemic status was induced.

## Materials and Methods

### Animal care and housing

Animal care was performed according to the guidelines established by the European Council Directive 2010/63/EU. Sixty female, SKH-2 brown phenotype hairless mice (34.4 ± 1.35 g), aged 13–14 weeks old, were bred and maintained in the animal care facility in the School of Pharmacy, National and Kapodistrian University of Athens (EL 25 BIO-06). The experimental procedure was approved by the Ethics Committee of Experimental Protocols, and permission was issued by the National Peripheral Veterinary Authority of Greece (Protocol Number: 718/11-02-2016).

### Diet preparation and administration

Female animals were randomly assigned to six groups of 10 animals each. They had unrestricted access to food and fresh water. The animal room was maintained at 23°C ± 1°C, 45% humidity and illuminated by white fluorescent tubes in a 12 h cycle of light and dark.

**FIG. 1.**
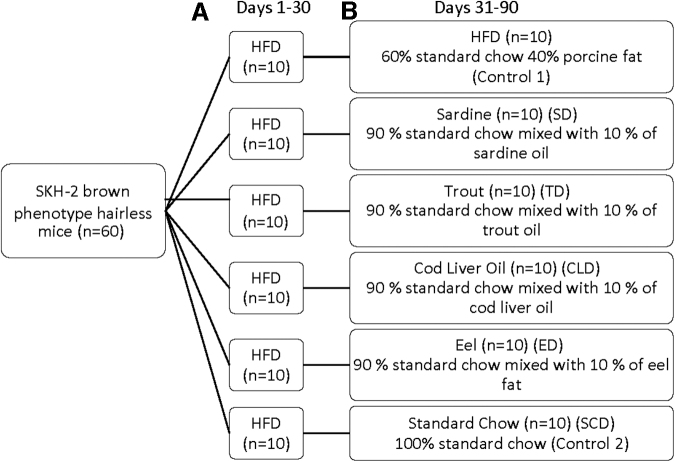
Experimental design and diet administration. All groups of animals were fed an HFD for 30 days **(A)**, after which they were transferred to the respective fat diets and fed for another 60 days **(B)**. HFD, high fat diet.

In the first 30 days of the experiment, all mice were fed exclusively with an HFD containing 60% standard chow (Nuevo S.A. Evia, Greece & Farma-Efyra, Korinthos Greece) and 40% pure porcine fat (Eilikrinia Butcher Company, Glyfada, Greece). For the subsequent 60 days, the six groups were administered the diets below (Fig. 1).

### Fish lipid preparation

Sardine and trout oils were extracted from 100 g of body remains after their industrial processing (KONVA, Kilkis, Greece and P. DIMOU, Thiva, Greece, respectively). After homogenization, the lipids were extracted by using a system of analytical-grade solvents containing chloroform, methanol, and water, in a ratio of 2/2/1. The mixture was filtered and, subsequently, the organic phase was collected. Thereafter, 1% sodium sulfate was added to absorb humidity and a second filtration was performed. Finally, the organic phase was transferred to a rotavapor (R-114/B-480 Buchi, Switzerland) and evaporated at 55°C to obtain the oil.

Eel lipids were extracted from 100 g of fatty residual that remained after a smoking process was applied to the fish by the aquaculture company V. GEITONAS & Co, Ioannina, Greece. The lipids were thereafter preserved at 2–4°C.

Cod liver oil used was the liquid version of the commercially available product “Seven Seas” Merck Group, Germany.

### Fish lipid analysis

The homogenized fish lipids were placed in a porous container allowing hexane to extract the lipid fraction continuously. The lipid content was determined by gas chromatography (GC) employing the Shimadzu GC system 2010 with a Shimadzu AOC-20i Auto-sampler equipped with a Shimadzu FID-2010 detector (Japan). The FA profile was performed by methylation and GC. GC conditions: BPx70 capillary column, 50 m length/0.33 mm dia., 0.25 mm film thickness; isothermally, 220°C oven temperature; 260°C detector temperature; carrier gas, He; and flow rate, 20 mL/min. with a 1/50 split. The reference standard was Supelco 37 Component FAME Mix, lot LRACO318 (Sigma Aldrich). Lipid analyses of each oil are represented in Table 1.

**Table 1.** Fish Oil Lipid Analysis of Homogenized Fish Tissue

Lipids	Sardine	Trout	Eel	Cod liver oil^a^
Saturated	47.6%	21.2%	31.9%	21.0
Monounsaturated FAs	31.6% (*ω*-9, 14.5%)	40.6% (*ω*-9, 36%)	38.1% (*ω*-9, 28.6%)	46.%
Polyunsaturated FAs	20.8% (*ω*-3, 17.5%; *ω*-6, 3.1%)	38.2% (*ω*-3, 6.9%); *ω*-6, 31.3%)	30.0% (*ω*-3, 13.2%; *ω*-6, 16.6%)	26.0% (*ω*-3, 20.6%; *ω*-6, 4.3%)
Total fat (g/100 g) of fish tissues	6.3%	14.7%	22.0%	Not known
ω-6/*ω*-3 ratio	0.18	4.54	1.26	0.21

^a^ Data listed on the tag of the commercial reference product “Seven Seas” (Merck).

FA, fatty acid.

### Blood lipid profile quantitation

Blood total cholesterol (TC), TGs, and HDL were measured by using a COBAS b 101 automatic analyzer (Roche, Basel, Switzerland). The blood was obtained from the mice's tail to fill the appropriate area of the test disk. The detection range given by the manufacturer is able to determine these biomarkers in mouse total blood (TC: 50–500, TG: 45–650, HDL: 15–100 mg/dL).

### Glucose measurement

The glucose determination was estimated electrochemically, by using Freestyle Precision Model test strips (Abbott Laboratories, Abbott Park, IL). A small blood drop, by performing a small cut on the mouse's tail, was collected for the respective measurement.^15^

### HDL measurement

In some cases, mice, especially those fed the HFD throughout the entire experimental period, exceeded the detection limits of the Cobas analyzer (100 mg/dL) and therefore HDL is expressed as a category that is dependent on approximate values rather than an absolute value. A statistical analysis of HDL was made on the following basis: HDL Category 0, (mg/dL) 0–25; Category 1, >25–50; Category II, >50–75; Category III, >75–99; and Category IV, >99–125.

### Statistical analysis

Data were analyzed by employing both a non-parametric and parametric methodology. The former used the Wilcoxon matched-pairs signed-ranks test, which overcomes the normal distribution assumption and is applicable for limited sample (<30) data points per variable. The latter employed the paired-samples *t*-test comparing the means of two measurements for the same diet group. Differences were considered as significant when *P* ≤ .05. The data in figures are expressed as mean ± standard deviation. Both tests provided similar results with regard to statistical significance, except in the case of the HFD comparison of days 1–30. The non-parametric method was reported for statistical analysis in this study, as the comparisons for each variable were made between day 30 and 90 values.

However, based on an earlier human study in which 10 volunteers who had received fish oil supplements exhibited a dramatic reduction in TG levels,^16^ we had assumed that a group size of 10 would be adequate for this study. Nevertheless, power calculations, using a *post hoc* calculator (ClinCalc.com) and testing between two means (day 30, hyperlipidemia status vs. day 90, the treatment effect on TGs), a sample size of 10 at (*P* = .05) provided a power of ≥0.80.

## Results

The eel was the fattiest fish tested (excluding cod liver oil) and it contained a large quantity of *ω*-3 FA, a moderate quantity of *ω*-6, and high levels of *ω*-9 FA. The trout was moderately fatty with minimal levels of *ω*-3 and the highest *ω*-6 and *ω*-9 FA levels. The sardine was the least fatty fish with a high concentration of *ω*-3 and the lowest levels of *ω*-6 and *ω*-9 FA.

With respect to the saturated and unsaturated lipids, the sardine contained the highest level of saturated FA, the trout the lowest, and the eel moderate levels as shown in Table 1.

### Days 1–30

**FIG. 2.**
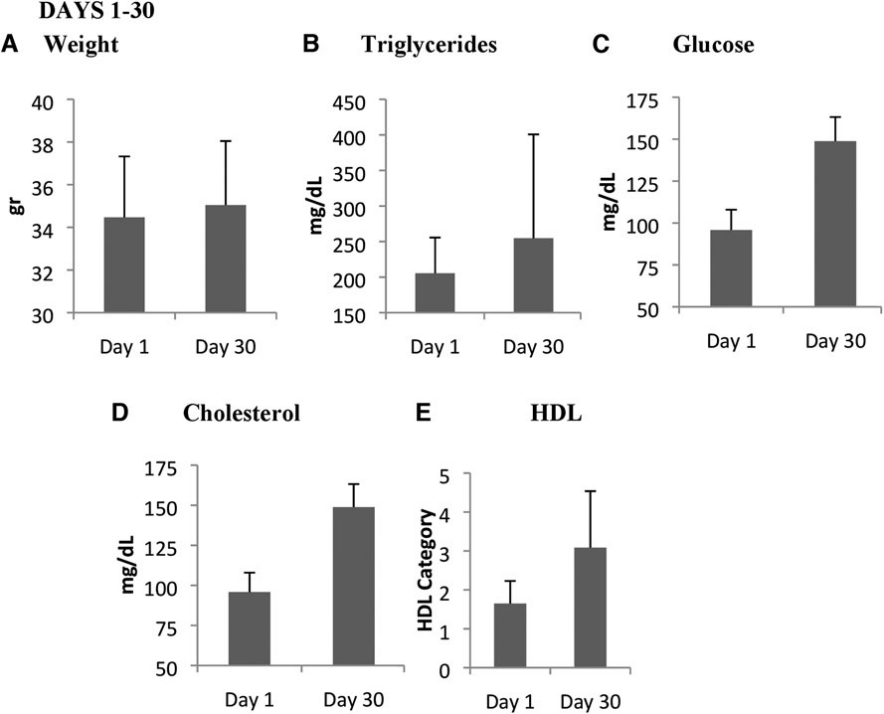
Comparison of weight **(A)**; TGs **(B)**; glucose **(C)**; cholesterol **(D)**; and HDL **(E)** at end of the 30 day HFD feeding period. All parameters at day 30 had increased significantly (*P* ≤ .05) compared with at day 1. HDL, high density lipoprotein; TG, triglyceride.

At the end of the HFD administration (day 30), the weight, TGs, glucose, cholesterol, and HDL demonstrated statistically significant (*P* ≤ .05) increases in comparison to the measurements of day 1. As seen in Figure 2, all of the dietary groups exhibited hyperlipidemic parameters.

### Days 31–90

**FIG. 3.**
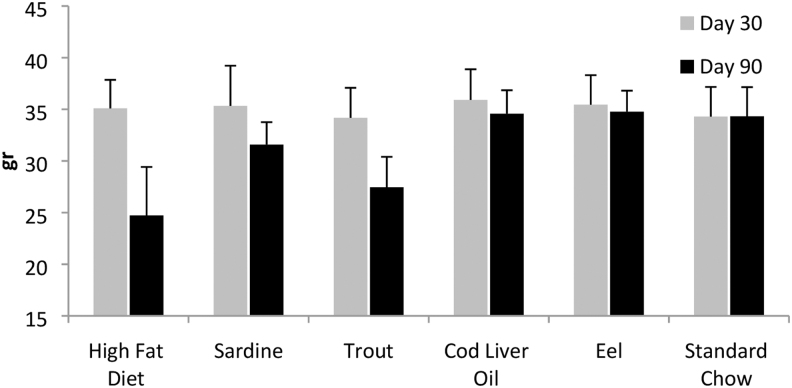
Body weight comparisons per respective diet. Weights increased significantly (*P* ≤ .05) for all diets except for animals receiving standard chow and eel oil during the treatment period (days 31–90).

The body weight decreased in a statistically significant manner in all diets (Fig. 3) during the treatment period (days 30–90), with the exception of the eel dietary group and the standard chow group (days 31–90).

As seen in Table 2, food intake remained about the same for all diets except for HFD and the trout oil diet, both of which declined dramatically.

**Table 2.** Average Daily Diet Consumption Per Mouse (*g*)

Diet	Days 31–60	Days 61–90
HFD	3.92	**0.9**
Sardine	4.62	4.2
Trout	2.55	**1.23**
Eel	4.34	4.85
Cod liver oil	3.85	4.11
Standard chow	4.00	4.00

Dietary intake of HFD and the trout oil group declined significantly (*P* < .05, bold values).

HFD, high fat diet.

The HFD animals demonstrated a marked decline in the consumption of food intake (3.92 g/day/mouse in days 30–60 vs. 0.9 g/day/mouse in days 61–90). At day 90, those animals that had been on the HFD throughout the experimental period were appearing unthrifty. It had been observed by Haven, in 1936, that animals receiving diets with 25% or more fat seldom survived.^17^ The loss of thriftiness in the HFD-fed animals may simply be a manifestation of lipid toxicity, as previously observed. Trout oil was composed of the highest *ω*-6 FA content and exhibited the highest *ω*-6/*ω*-3 ratio, 3.5 times that of Eel oil.

**FIG. 4.**
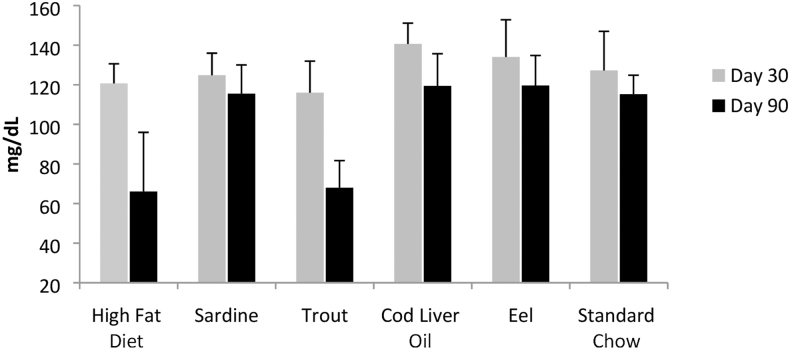
Blood glucose levels. All dietary groups, with the exception of sardine and standard chow, exhibited a significant (*P* ≤ .05) decrease in blood glucose level at the end of the treatment period.

All diets, except the sardine and standard chow, exhibited a significant decrease in blood glucose levels (Fig. 4).

**FIG. 5.**
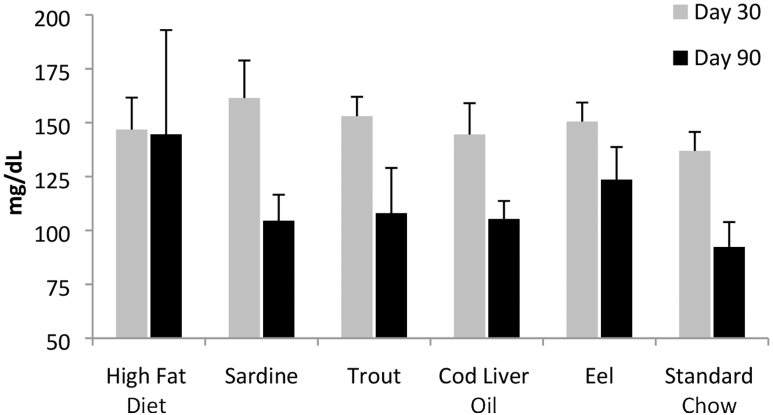
Cholesterol levels. Cholesterol levels decreased in all diets except the HFD.

Cholesterol levels decreased in all diets except the HFD (Fig. 5). The shift from HFD to normal (standard chow) or fish lipid diets broadly re-established normal cholesterol levels.

**FIG. 6.**
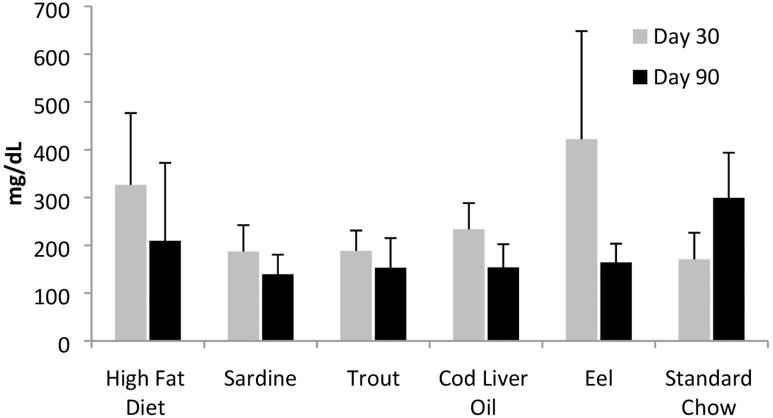
TG levels. At the end of the dietary treatment period, TG levels in eel and cod liver oil diets decreased significantly (*P* ≤ .05) whereas the standard chow group exhibited a significant increase in TG level.

The eel and the cod liver oil groups demonstrated a statistically significant decrease in terms of TGs, whereas the standard chow showed a statistically significant increase (Fig. 6). The reduction, in the case of the eel oil, was greatest among all dietary groups.

**FIG. 7.**
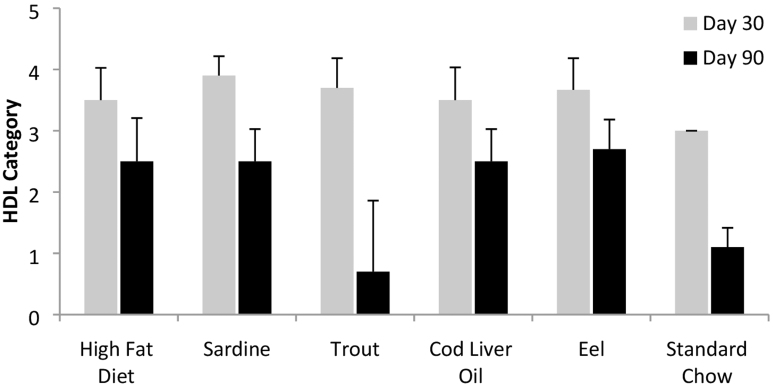
HDL levels. Although HDL levels decreased in all diets, only the decreases in the trout and standard chow groups were statistically significant (*P* ≤ .05).

HDL levels decreased by day 90 in all dietary groups (Fig. 7). The trout group showed the steepest drop, followed by the standard chow group. These decreases were statistically significant (*P* ≤ .05). None of the other diets demonstrated a statistically significant drop, although all other groups exhibited marked declines.

Cod liver oil exhibited the highest composition in total *ω*-3 FA (20.6%), followed by eel, which demonstrated an *ω*-3 FA content at 13.2% (Table 1). Sardine's lipid composition showed the lowest amount of *ω*-6 FA (3.1%), whereas *ω*-3 FA were abundant (17.5%). Sardine oil exhibited the lowest *ω*-6/*ω*-3 ratio (0.15). Trout had a *ω*-3 FA content of 6.9% and the highest *ω*-6 content with an *ω*-6/*ω*-3 ratio of 4.53.

The variation and complexity of the composition of the oils tested make relative ordering of their effect on ameliorating the HFD-induced hyperlipidemia difficult. All the oils displayed a similar composition regarding both monounsaturated FAs (30–40%) and trans lipids (1–2.2%, not shown) (Table 1). The PUFA to saturated FA (P/S) ratio was lowest with sardine oil (0.43), followed by eel (0.94), cod liver (1.21), and trout oil (1.82). The high levels of vitamins A and D listed in cod liver oil are another confounding variable. The omega-6/omega-3 ratios ranged from sardine (0.15), trout, (4.53), eel (1.3), and cod liver, (0.21). Despite the variation and complexity of the composition of the fish oils tested, the relative ordering of their effect on mixed dyslipidemia (high cholesterol and TGs) was eel ≤ cod liver oil. Their effect was most likely related to their high *ω*-3 FA content. Eel oil exhibited an advantage that was probably due to its *ω*-6/*ω*-3 ratio of near unity. Both eel and cod liver oils also significantly (*P* ≤ .05) reduced blood glucose levels by the end of the treatment period. Trout oil significantly reduced glucose, cholesterol, and HDL levels; whereas sardine oil, with a high level of *ω*-3 FA and a low *ω*-6/*ω*-3 ratio, only reduced cholesterol levels significantly (*P* ≤ .05). Overall, smoked eel was found to be the best source of *ω*-3 FA, with a balance of *ω*-6 FA, that ameliorated HFD-induced mixed hyperlipidemia.

## Discussion

Hyperlipidemia is a major risk factor for both CVD and diabetes.^18,19^ Both are participant risk factors for metabolic syndrome (MS), an important public health challenge worldwide.^20^ As lifestyle modifications are routinely recommended to reduce CVD risk, nutritional factors play an important role in addressing aberrant lipid metabolism and maintaining a healthy lipid profile.^18,21^

Although the exact mechanism of action of *ω*-3 FA in promoting health benefits is unknown, two theories have been developed to address this issue. One theory has recently developed from studies of DHA from fish oil and that has shown benefits in treating MS symptoms. Molecular recognition studies of DHA have shown that this *ω*-3 FA has high affinity for peroxisome proliferator-activated receptors and retinoid-X receptor *α*.^22^ The affinity produces effects on the first four carbons of the FA and it is suggested that the beneficial effects may be produced by this high affinity.

Moreover, the most convincing theory, thus far, is one involving inflammatory reactions. It is known that *ω*-3 FA are anti-inflammatory and anti-arrhythmic. Indeed, the seminal observations on the relationship of diets high in omega-3 FA reported that these FA were associated with low incidence of ischemic heart disease and inflammatory symptoms.^23,24^

Studies with the Skh mouse have shown that approximately equal levels of 18:3 FA were present in the plasma and epidermis of animals fed either omega-3 or omega-6 FA diets, suggesting that Δ^6^-desaturase activity was not inhibited by omega-3 FA.^25^ There were differences, however, in the levels of 20:3 and 20:4 FA, with threefold higher levels in animals fed an omega-6 FA diet. There was a consistent reduction in these FA in animals fed the omega-3 FA diet. This consistent reduction pointed to a possible inhibitory effect on elongase, Δ^5^-desaturase, or both. An inhibitory effect on the desaturase was supported by an observed decrease in the 20:4/20:3 ratio. Reduced levels of 20:4 FA in the omega-3 FA diet could be rate-limiting with respect to eicosanoid metabolism. A summary of the anti-inflammatory effects of *ω*-3 FA has been discussed^26^ and includes decreased levels of pro-inflammatory and immunosuppressive prostaglandin E2 (PGE2)^27^; *ω*-3 FA modulate a number of cytokines and prostaglandins that mediate inflammatory and immune responses^28^; *ω*-3 FA inhibits certain UV-induced genotoxic markers, such as cutaneous p53 expression^29^; and *ω*-3 FA increase the erythema (sunburn, an inflammatory response) threshold to UV-irradiation.^16^ Most of these responses result from a competition of *ω*-6 and *ω*-3 FA for active sites on the cyclooxygenase enzyme complex, and omega-3 FA-mediated reduced 20:4 FA levels, through which the pro-inflammatory and immunosuppressive intermediates are synthesized. Nevertheless, the balance between *ω*-6 and *ω*-3 FA is “an important determinant in decreasing the risk for coronary disease”, both in the primary and secondary prevention of coronary disease.^30^

Regardless of mechanism, the current study supports the role of *ω*-3 FA in ameliorating the hyperlipidemia induced by an HFD. It also suggests that further investigations will be necessary to select dietary sources, such as eel, that will be most effective in managing dyslipidemia and maintaining a healthy lipid profile.
